# Assessment of Human Multi-Potent Hematopoietic Stem/Progenitor Cell Potential Using a Single *In Vitro* Screening System

**DOI:** 10.1371/journal.pone.0050495

**Published:** 2012-11-28

**Authors:** Julien Calvo, Aissa BenYoucef, Jan Baijer, Marie-Christine Rouyez, Francoise Pflumio

**Affiliations:** 1 CEA, Laboratoire des Cellules Souches Hématopoïétiques et Leucémiques, Institut de Radiobiologie Cellulaire et Moléculaire (IRCM), Fontenay-aux-Roses, France; 2 INSERM, UMR 967, Fontenay-aux-Roses, France; 3 Université Paris Diderot, UMR 967, Sorbonne Paris Cité, Fontenay-aux-Roses, France; 4 Université Paris-Sud, UMR 967, Fontenay-aux-Roses, France; 5 CEA, IRCM, Cytometry Service, Fontenay-aux-Roses, France; 6 INSERM, U1016, Paris, France; University of Sao Paulo - USP, Brazil

## Abstract

Hematopoietic stem cells are responsible for the generation of the entire blood system through life. This characteristic relies on their ability to self renew and on their multi-potentiality. Thus quantification of the number of hematopoietic stem cells in a given cell population requires to show both properties in the studied cell populations. Although xenografts models that support human hematopoietic stem cells have been described, such *in vivo* experimental systems remain restrictive for high throughput screening purposes for example. In this work we developed a conditional tetracycline inducible system controlling the expression of the human NOTCH ligand Delta-like 1 in the murine stromal MS5 cells. We cultured hematopoietic immature cells enriched in progenitor/stem cells in contact with MS5 cells that conditionally express Delta-like 1, in conditions designed to generate multipotential lineage differentiation. We show that upon induction or repression of DL1 expression during co-culture, human immature CD34^+^CD38^−/low^(CD45RA^−^CD90^+^) cells can express their B, T, NK, granulo/monocytic and erythroid potentials in a single well, and at the single cell level. We also document the interference of low NOTCH activation with human B and myelo/erythroid lymphoid differentiation. This system represents a novel tool to precisely quantify human hematopoietic immature cells with both lymphoid and myeloid potentials.

## Introduction

The hematopoietic system originates from the proliferation and differentiation of a rare population of cells named the hematopoietic stem cell (HSC). During development, HSC are located in different environments, from the aorta-gonade-mesonephros area in embryos, through the foetal liver in foetuses to the bone marrow (BM) in adults. These different niches control the balance of quiescence and divisions of HSC allowing them to arise, proliferate, maintain and generate the large variety of mature blood cells [1].

Studying HSC requires sophisticated experimental systems that assay their fundamental properties, including self-renewal and multi-potentiality. The most conventional way to study these primitive cells is to serially transplant a given cell population into irradiated compatible mouse recipients [2]. Although essential, this assay remains restrictive and costly. It necessitates the manipulation and housing of tolerant animals as well as specific facilities such as an irradiation unit. Studying human HSC is even more complicated as it requires developing xenografts models using immune-deficient mice that are highly sensitive to infections [3]. *In vitro* culture systems have been described that assay specific differentiation programs from primitive human cells [4]. These assays have been very powerful to study the development of dedicated lineages; however when it comes to study multi-potentiality, such systems are not useful anymore, as they can be mutually exclusive because of activation of specific molecular pathways. For instance T cell development that normally takes place in the thymus and requires specific protein interactions, such as a NOTCH/Delta-like ¼ (DL1, DL4) signalling pathway activation [5], is not permissive to B cell differentiation [6], [7]. Thus combining all hematopoietic differentiations into a single assay is a difficult task. We have previously shown that multi-potential development from single human primitive cells from cord blood (CB) was possible *in vitro*. However such approach requires to initiate cultures on a mouse stromal feeder, for instance MS5 cells, and later to split proliferating clones into two independent cultures, MS5 co-cultures [8] and Fetal-thymic-organ-culture (FTOC) [9], to circumvent different human exclusive lineages [10]. Another strategy proposed to mix two stromal feeders that express or not the NOTCH ligand DL4 [11] or to culture cells in presence of low levels of inhibitors of NOTCH, such as gamma secretase inhibitors (GSI) [12], in order to drive multiple differentiations from cultures of single defined human cells. Important caveats of these approaches include the potential overgrowth of one of the two cell lines or off-target effects of GSI.

**Figure 1:**
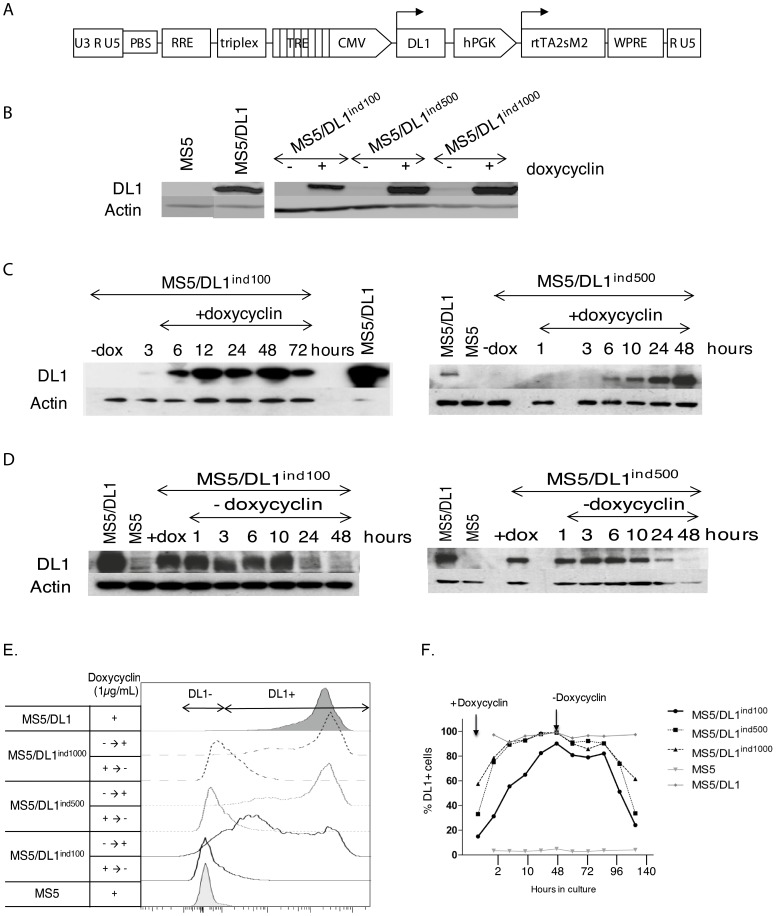
Characterization of MS5/DL1^ind^ cells lines. (A) Schematic representation of the TET/on lentiviral DL1 vector system. (B–D) Measure of DL1 expression in established MS5 cells. (B) Cell lines, previously transduced with 100 (/DL1^ind100^), 500 (/DL1^ind500^) or 1000 (/DL1^ind1000^) ng P24 vectors, were cultured in presence (+) or absence (−) of 1 µg/ml of doxycyclin during 48 hours. Proteins were extracted and DL1 expression was analysed by western blot. (C) Follow up of DL1 induction. MS5/DL1^ind100–500^ cells cultured with (+) or without (−) doxycyclin (1 µg/ml) were lysed at different times (in hours) after adding doxycyclin to the culture medium. (D) Follow up of DL1 drop down expression according to time after induction. DL1 expression was induced 24 hours before washing out medium (time 0 hour). At different time points, cells were harvested and protein extracted to follow up decrease of DL1 expression. (E) Surface expression of human DL1 in MS5 cell lines detected by flow cytometry. Shown are histograms of DL1 expression levels in presence of 1 µg/mL doxycycline (+ condition), after 48 hours of induction (- ->+condition) and 72 hours after washing out doxycycline from the medium (+ -> - condition). Arrows indicate positive and negative DL1 expression on cells. (F) Follow up on DL1 surface expression as a function of time after adding (+ doxycyclin) or washing out (-doxycyclin) doxycycline in the culture. Shown are % of DL1^+^ cells measured as in (E).

In the present work, we developed MS5 stromal cells expressing conditional tetracycline (TET/on) inducible DL1 (MS5/DL1^ind^). Upon addition (or removal) of doxycyclin in the culture medium, we could reproducibly induce (or suppress) DL1 protein expression from MS5/DL1^ind^ cells. We show human T cell development from CD34^+^CD38^−/low^ (CD45RA^−^CD90^+^) CB cells using the MS5/DL1^ind^ cell lines developed, comparable to MS5/DL1 cells that can sustain normal human T cell development from circulating CD34^+^CD7^−^ cells [13] as well as proliferation of human T-ALL [13], [14]. Using the conditional (on/off) expression of DL1, we further describe novel and simple experimental conditions that allow human hematopoietic primitive multi-potent cells to differentiate into multiple T, B, Natural Killer (NK), granulo-monocytic (G/M) and erythroid lineages.

**Figure 2:**
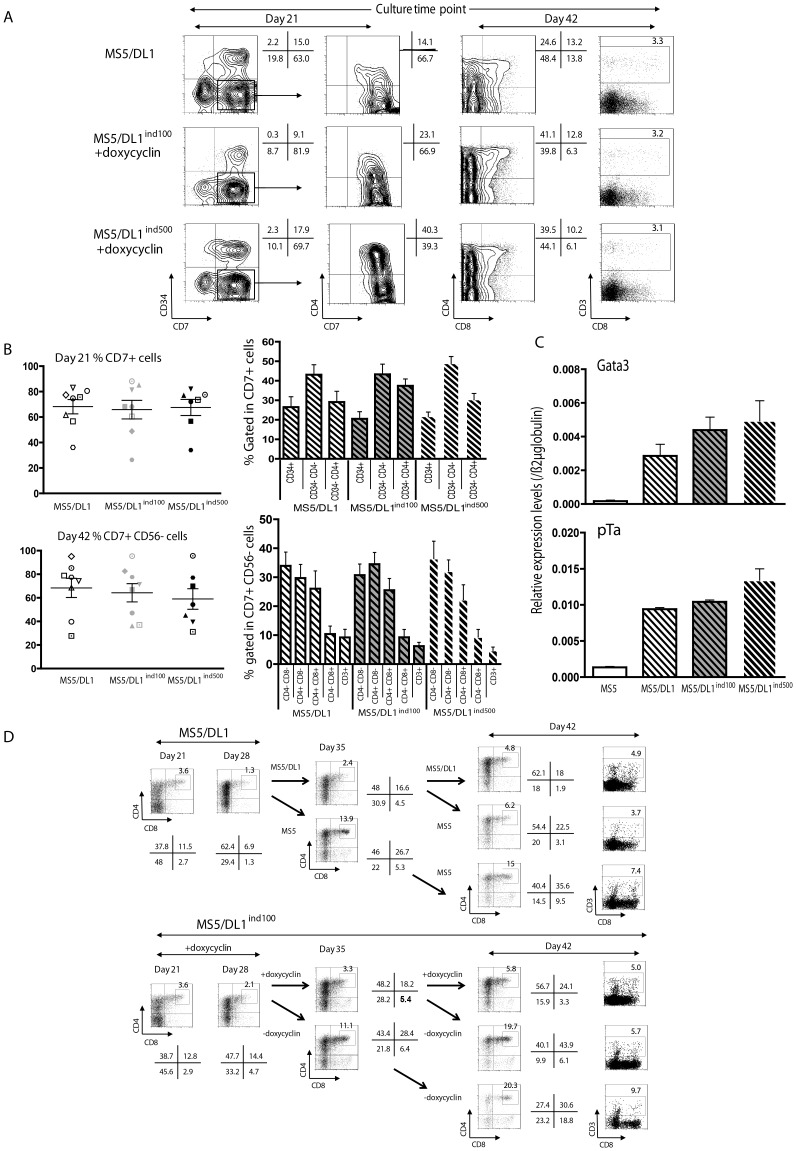
T cell differentiation from CD34^+^CD38^−/low^ cells cultured with MS5 cell lines in presence of 1 µg/ml doxycyclin. (A) 5000 cells were cultured in complete medium with MS5/DL1 and MS5/DL1^ind^ cell lines. Flow cytometry was performed on the progeny of such cells 21 and 42 days after initiating the cultures. Shown are results of labelled cells with anti-human specific antibodies directed against CD34, CD7, CD4, CD8 and CD3. % of positive cells are indicated beside each quadrant. (B) Summary results of analysing the progeny of 5–10×10^3^ CD34^+^CD38^−/low^ cells from 7–8 individual CB samples. Left: shown are % of CD7^+^ cells and every symbols represent an individual sample. Right, Proportion of indicated populations gated in the CD7^+^ cells for every time point tested. Shown are mean +/− SEM (C) Levels of NOTCH target gene transcripts at 21 days of culture with doxycyclin. Results were normalized over ß2m transcripts levels for each sample. (D) Follow up of T cell differentiation after switching DL1 expression off (MS5 or MS5/DL1^ind^ – doxycyclin) during culture. Shown are results from 1 out of 2 experiments. Flow cytometry was performed at 3 time points for the measure of CD4 and CD8 expression on human cells. % of positive cells are indicated beside every dot plot. When a specific gate was drawn, % of cells is indicated beside the gate. K&W statistical analysis was used for data of this figure.

**Figure 3:**
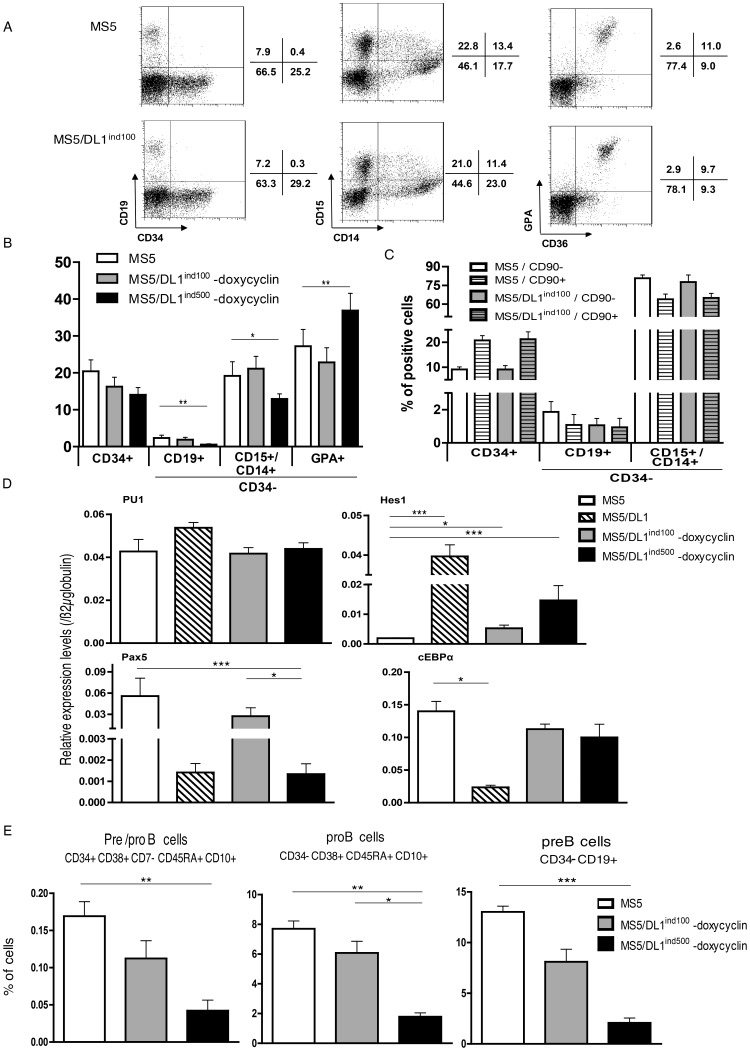
Human B, myeloid and erythroid development from CD34^+^CD38^−/low^(CD45RA^+^CD90^+/−^) cells cultured with MS5 and MS5/DL1^ind^ cells. 5×10^3^ CD34^+^CD38^−/low^(CD45RA^+^CD90^+/−^) cells were cultured in absence of DL1 expression (MS5 or – doxycyclin) during 21 days. Cells were harvested and analysed for the expression of CD19 (B cells), CD14 and CD15 (Myeloid cells) and CD36/GPA (erythroid cells). (A) Shown are results from a representative experiment performed with CD34^+^CD38^−/low^ cells. (B) Summary of results of cultures of 1–5×10^3^ CD34^+^CD38^−/low^ cells from 3–5 CB samples. (C) Results from cultures of 1–5×10^3^ CD34^+^CD38^−/low^CD45RA^−^CD90^+^ or CD90^−^ cells. Shown of mean % of cells obtained with 3 CB samples. (D) Transcripts levels in CD34^+^CD38^−/low^ cells cultured 21 days with MS5 cell lines in absence of doxycyclin. Results are normalized over ß2m expression levels. (E) Characterization of B cell progenitor populations generated during cultures of 10×10^3^ CD34^+^CD38^−/low^ cells and harvested at 21 days. Shown are means of 2 experiments performed with distinct CB samples. M&W and K&W statistics were respectively used for data in (C) and (B, D, E).

**Figure 4:**
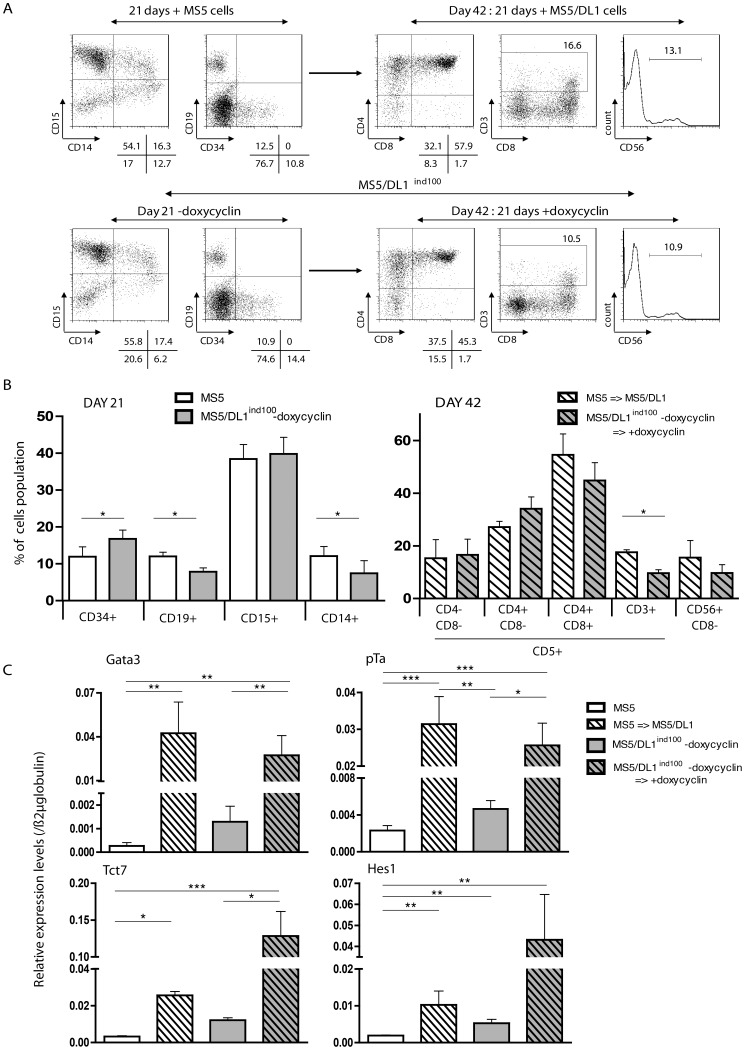
Multi-lineage differentiation of human CD34^+^CD38^−/low^ cells in co-cultures with MS5 cell lines. Sorted cells (10×10^3^ cells/well) were cultured 21 days without DL1/expressing stromal cells (MS5 or MS5/DL1^ind100^–doxycyclin). Cells were harvested and analysed by FACS for the presence of myeloid (CD14^+^/CD15^+^), lymphoid B (CD19^+^) and progenitor (CD34^+^) cells. Half of cells were plated with DL1/expressing stromal cells (MS5/DL1 or MS5/DL1^ind100^+ doxycyclin). (A) Results of a representative experiment. % of expressing cells are indicated under the plots in case of quadrant stat or beside the gated population. (B) Summary results of 3 CB samples. (C) Levels of *Gata3*, *pT*α, *TCF7* and *Hes1* transcripts in the progeny of CD34^+^CD38^−/low^ cells before (MS5 or MS5/DL1^ind100^–doxycyclin) and after (MS5/DL1 or MS5/DL1^ind100^+doxycyclin) DL1 induction during culture. Results were normalized over ß2m transcript levels. M&W and K&W statistical analyses were respectively used in (B) and (C).

**Figure 5:**
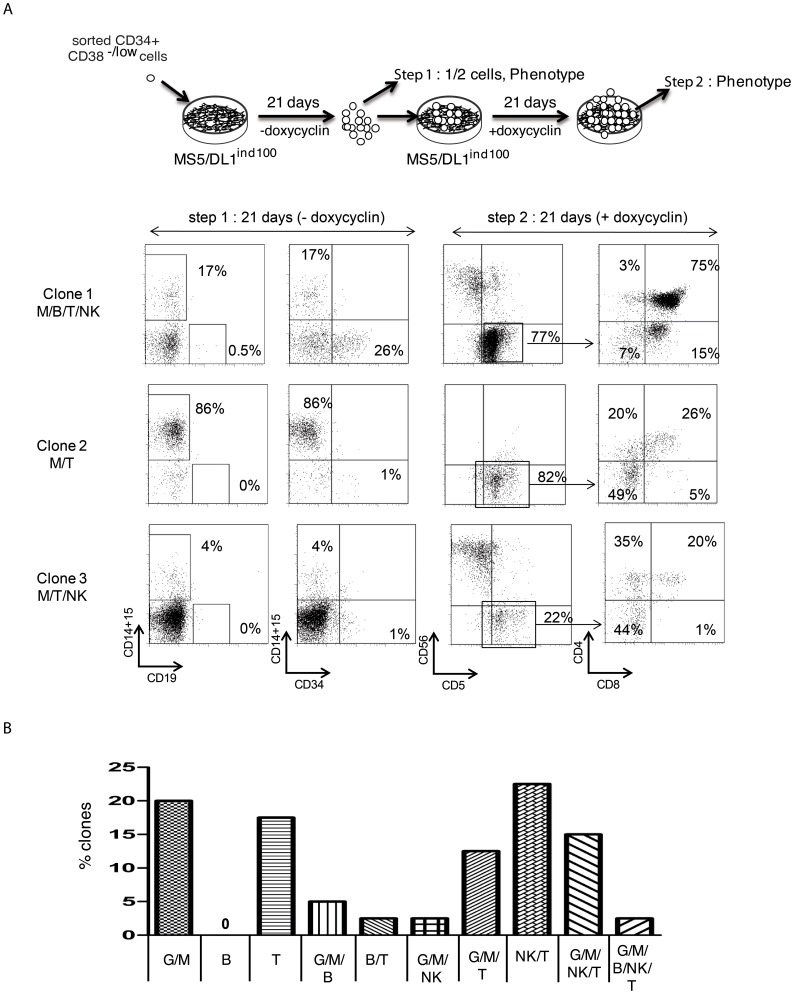
Single cell cultures of CD34^+^CD38^−/low^ cells with MS5/DL1^ind100^ cells. (A) Experimental design and analysis of 3 representative clones by FACS before (step 1: MS5/DL1^ind100^–doxycyclin, day 21) and after (step 2: MS5/DL1^ind100^+doxycyclin, day 42) induction of DL1 during culture. G/M, CD14^+^/CD15^+^; B, CD19^+^; T, CD5^+^CD4^+^CD8^+/^; NK, CD56^+^CD5^−^. (B) Distribution of different clones obtained in the culture of 180 CD34^+^CD38^−/low^ cells, among which 57 proliferated enough to allow FACS analysis at 21 days. Indicated is the proportion of different potentials measured in the proliferating clones.

## Methods

### Human CB Cells

CB samples were collected from healthy infants with the informed written consent of the mothers. CB samplings and experiments described here were done in accordance with a signed authorization from Clinique des Noriets (Vitry-sur-Seine, France) advisory board and with a signed authorization between Agence Régionale de Santé and Hôpital Antoine Beclère (Clamart, France). CD34^+^ cells were purified by immunomagnetic selection using a CD34 MicroBeads kit (Myltenyi Biotec, Paris, France). CD34^+^CD38^−/low^(CD45RA^−^CD90^+^) cells were sorted after labeling with monoclonal antibodies (MoAbs) directed against CD38 (clone T16), CD45RA (ABL11), CD34 (581) coupled to fluorescein (FITC), phycoerythrin (PE), PE-cyanin 7 (Pc7) or allophycocyanin (APC) (Beckman Coulter, Villepinte, France) and CD90 (5E10) (Becton Dickinson (BD), Le Pont de Claix, France). Sorting was performed using an INFLUX cell sorter (BD, France).

### Vector Constructs

pΔ500Trip-TRE-Tight-huDL1-huPGK-rtTA2-M2-WPRE inducible (pV81/DL1^ind^) vector was constructed after a series of cloning steps. Briefly, pΔ500Trip-PGK-rtTA2-M2-Ins-CMVmin-Luciferase-WPRE self-inactivating vector (kindly provided by Dr R. Vogel, CNRS-UMR7091, Paris, France) was digested with XhoI-BamHI and the pTREtight promoter from Clontech (Montain View, CA) was inserted to replace the tetracycline-regulated minimal CMV (CMVmin) promoter. Luciferase was eliminated by a partial BAMH1-Nde1 digestion and replaced by a polylinker (MS- forward : 5′-TATGGATCCAGTTAACCTGCAGGTCGACC-3′ and oligonucleotide reverse : 5′-GATCGGTCGACCTGCAGGTTAACTGGATCCA-3′) containing a Sal1 restriction site. Human DL1 sequence was recovered after a partial BamHI-XhoI digestion of pTRIP/ΔU3-EF1α-DL1-IRES-GFP vector [14] and ligated into BamH1-Sal1 digested pΔ500Trip-TRE-Tight-MS-huPGK-rtTA2-M2-WPRE allowing obtaining the pV81/DL1^ind^ vector (Figure 1A). The human *dl1* cDNA was originally kindly provided by Dr E Parreira, Gulbenkian Instituto, Lisboa, Portugal [15]. Lentiviral vectors were produced as previously reported [16].

### MS5 Cells

Mouse stromal MS5 cells were originally obtained from Dr K Mori (Nagata University, Japan). MS5/DL1 cells have been described in [14]. For inducible DL1 expression, MS5 cells were transduced using different PV81/DL1^ind^ vector concentrations calculated according to P24 protein detection by ELISA (Cell Biolabs/Euromedex, Mundolsheim, France). The MS5/DL1^ind100^,/DL1^ind500^ and DL1/^ind1000^ cell lines used in this study were obtained after transduction of MS5 cells with respectively 100 ng, 500 ng and 1000 ng P24 virus titer/5×10^4^ cells and expansion of the transduced cells.

### T Cell Cultures

Sorted CD34^+^CD38^−/low^(CD45RA^−^CD90^+^) cells (1–15.10^3^/well, detailed in figure legends) were co-cultured in contact with MS5/DL1 or MS5/DL1^ind^ cells (2.8.10^4^ cells/cm^2^) in reconstituted alpha-MEM supplemented with 10% FCS (06450, StemCell Technologies, Grenoble, France) and 10% human AB serum (J Boy, Reims, France), in presence of recombinant human stem cell factor (50 ng/ml, Amgen, Neuilly-sur-Seine, France), rhFlt3-ligand (20 ng/ml, Diaclone, Besançon, France), Insulin (20 nM, Sigma-Aldrich, St Louis, MO) and rhIL-7 (10 ng/ml, R&D System, Minneapolis, MN). Medium was half changed twice a week and every stromal layer was renewed once a week. At passage time point, hematopoietic cells were counted and 100 µL containing cells were labelled with specific anti-human antibodies when enough cells were available for FACS analysis. Doxycyclin (1 µg/ml, Sigma-Aldrich, MO) was added at every medium renewal. Upon removal of Doxycyclin, wells were carefully washed using phosphate buffer saline (PBS) (14090, Invitrogen, France) and fresh medium w/o doxycyclin was added. This step was done on days of co-cultures passage time.

### Multipotential T, B, E and M/G Cultures

Sorted hematopoietic cells were co-cultured with pre-established MS5 cells in medium (as described in T cell cultures) supplemented or not with erythropoietin (EPO, 2 U/mL, 02625, StemCell Technologies, France) during 21 days with weekly half-changed medium. Cells were then harvested and processed for FACS analysis.

In case of testing T cell potential in the same cultures, at 21 days, harvested cells were split into two, half being used for FACS analysis. The remaining half-cell content was re-seeded on MS5/DL1 or MS5/DL1^ind^ cells with fresh medium supplemented with doxycyclin. T cell cultures proceeded then as described previously.

For clonal cultures, individual cells were sorted in 96 well plates in 50 µl of complete medium. Clonal cultures were not passaged, and medium was refreshed three times a week (retrieval of 30 µl and addition of 50 µl/well). Wells in which proliferating clones were detected by microscopy 3 to 4 weeks after initiation of cultures were individually harvested after adding 50 µl of trypsin/well. Half of individual clones was used for phenotyping by flow cytometry and the remaining half was spin down, res-suspended in 50 µL of fresh medium containing Doxycyclin and re-seeded in 96 well/plates. Cultures were continued 21 days with refreshment of medium three times a week. At 42 to 49 days, cells were harvested and labelled with antibodies for flow cytometry analysis.

### Flow Cytometry

Immuno-phenotyping of culture-derived human cells was performed on a FACSCalibur and on a LSR-II flow cytometer (BD, France) using PE-, PC7-, APC- and FITC-conjugated mouse anti-human specific monoclonal antibodies CD45 (J.33), CD34 (581), CD15 (80H5), CD19 (J4.119), CD4 (13B8.2), CD3 (UCHT1), CD8 (B9.11), CD7 (8H8.1), CD38 (T16), CD45RA (ABL11), CD10 (SN5c). All antibodies were from Beckman Coulter and from eBiosciences (Paris, France), including isotype controls.

Measurement of human DL1 surface expression in MS5 cell lines was done using the anti-human DL1 (clone MHD1–314) coupled to APC (Myltenyi Biotec).

### Western Blotting

DL1 expression was analysed using 10 µg of proteins obtained from MS5-DL1 or MS5/DL1^ind^ cells in western blot analysis [14]. Antibodies were against human DL1 (C20, Santa Cruz Biotechnology, Santa Cruz, CA) and β-actine (AC-15, Sigma-Aldrich, France).

### Gene Expression Analysis

Quantitative polymerase chain reaction (Q-PCR) analysis was performed using the 7900HT Fast Real-Time PCR System (Applied Biosystems, Villebon sur Yvette, France). Reactions were performed in 20 µL volume with 0.5 µM primers and power SYBER green PCR master mix (4367660, Applied Biosystems) including SYBR® Green 1 Dye, nucleotides, AmpliTaq Gold DNA polymerase and optimized buffer components. Typical Q-PCR consisted in a AmpliTag Gold polymerase activation step at 95°C for 10 minutes followed by 40 cycles containing 2 steps. First step 95°C for 15 seconds and the elongation step at 60°C for 1 minute, followed by a 60°C to 95°C dissociation step every 15 seconds. Primers were designed to span introns and not to blast with murine genome. Primers were tested on dilution of cDNA from CD34^+^ cells to test PCR efficiency and specificity. Sequences of primers are*: β2microglobulin* (*β2* *m*) sense: CACAGCCCAAGATAGTTAAGT; antisense: CCAGCCCTCCTAGAGC; *hairy and enhancer of split 1* (*hes1*), sense : CAACACGACACCGGATAAACC; antisense : CCAGAATGTCCGCCTTC; *pre T-cell antigen receptor alpha* (*pTα*), sense : GTGTCCAGCCCTACCCA; antisense : ATCCACCAGCAGCATGATTG; *deltex1*, sense : TTCTGACTTCAGGAGCGAAAG; antisense : TGCCCACTCCCAACGA; *Gata3*, sense : CTCTCTGCTCTTCGCTACCC; antisense : GCGACGACTCTGCAATTCT; *receptor Interleukin 7* (*IL7r*), sense : GCTTTTGAGGACCCAGATGT; antisense : AGGCACTTTACCTCCACGAG; *paired box gene 5* (*pax5*), sense : CAGGCAGCTTCGGGTCAG; antisense : GGCGTTTATATTCAGCGATTT; *CCAAT/enhancer binding protein alpha* (*cEBPα*), sense : ATTGCCTAGGAACACGAAGC; antisense : GCACAGAGGCCAGATACA; *spleen focus forming virus (SFFV) proviral integration oncogene spi1* (*spi1*), sense : TGGATGTTACAGGCGTGCAA; antisense : GCGTTTGGCGTTGGTATAGAT; *transcription factor 7* (*TCF7*), sense : CTTCGACCGCAACCTGAA; antisense : TTGATGGTTGGCTTCTTGG. Each sample data were normalized over *β2* *m* values.

### Statistical Analysis

Two non-parametric statistical analyses, Mann and Whitney (M&W) and Kruskal and Wallis (K&W), were used to determine significance of the data. *, p<0.05; **, p<0.01; ***, p<0.001.

## Results

### Modulation of DL1 Expression in MS5/DL1^ind^ Cells

MS5 cell lines were cultured in presence and absence of 1 µg/ml doxycyclin and DL1 levels were measured. Results indicate that MS5/DL1^ind^ cells consistently up-regulate DL1 expression in presence of doxycyclin (Figure1B-D). Western blot analysis showed that total levels of DL1 were lower in doxycyclin treated MS5/DL1^ind100–500^ cells than in MS5/DL1 and in MS5/DL1^ind1000^ cells. Interestingly, surface expression of DL1 measured by flow cytometry showed that MS5/DL1^ind100^ cells had heterogeneous levels of DL1 expression compared to MS5/DL1^ind500–1000^ and MS5/DL1 cells (Figure 1E). Without doxycyclin, leakage of DL1 expression levels was mainly detected in MS5/DL1^ind500–1000^ cells (Figure 1B, 1F). DL1 expression was induced within a few hours of doxycyclin treatment and remained high for 2–3 days without doxycyclin re-feeding of cultures; compatible with the fact that doxycyclin is still present during this period (Figure 1C, 1F). Inversely, DL1 expression progressively decreased after washing out doxycyclin from the culture medium (Figure 1D, 1F). These results indicate that DL1 expression levels can be rapidly switched on and off in MS5/DL1^ind^ cells as a function of doxycyclin levels.

### T Cell Development from Co-cultures with MS5/DL1 and MS5/DL1^ind^ Cells

Sorted CD34^+^CD38^−/low^ CB cells were co-cultured with MS5/DL1, MS5/DL1^ind100^ and MS5/DL1^ind500^ cells in presence of doxycyclin. Human cells generated at 21 and 42 days were characterized. Results indicate that MS5/DL1 cells allow human T cell development (Figure 2A), although less efficiently than OP9/DL1 cells (Figure S1) [17]. MS5/DL1^ind100^ and MS5/DL1^ind500^ cells were also permissive to human T cell development as pro/CD34^+^CD7^+^ and pre/CD34^−^CD7^+^CD4^+/−^ T cells were reproducibly detected in the culture at early (21 days) time points (Figure 2A and B). Progression toward more mature CD4^+^CD8^+^ double positive (DP) and CD3^+^ T cells was observed at 42 days of culture whatever MS5 cell line tested (Figure 2A and B). T cell differentiation from 6 individual CB samples did not significantly differ between the three stromal cell lines, i.e. MS5/DL1 and MS5/DL1^ind100–500^ (Figure 2B and Figure S2). Similar results were obtained when CD34^+^CD38^−/low^CD45RA^−^CD90^+^ HSC-enriched cells from 3 CB were cultured (Figure S3) [18]. CD56^+^CD8^−^ NK cells were also detected in these cultures, with a high variability between CB samples (Figure S4). As for T cells, no significant difference in promoting NK cell development was observed between stromal lines. Measurement of Gata3 and pTα transcript levels at 21 days of culture with doxycyclin showed comparable levels of expression in cells recovered from the different conditions (Figure 2C).

T cell progenitors evolve in different areas of the thymus where they interact with thymic stromal cells and proteins that are critical for the progression of T cell maturation. For example, early T cell development is strongly dependent on NOTCH signalling whereas progression through later stages, such as the generation of DP cells, requires down regulation of NOTCH signalling [12]. In order to mimic T cell development, we switched off NOTCH signalling in cultures at different time points and followed up the development of DP cells. Results indicate that switching from MS5/DL1 to MS5 cells co-cultures or washing out doxycyclin from MS5/DL1^ind100^ or MS5/DL1^ind500^ cells co-cultures (Figure 1D) led to a similar improvement in T cell maturation, through more DP cell generation (Figure 2D and Figure S5). In NOTCH/off condition, a population of DP^high^ developed as well as CD3^+^ cells. However, fewer cells were recovered from the NOTCH/off vs NOTCH/on condition in relation with a decrease of proliferation (Figure S6). Altogether these results show that MS5/DL1^ind^ cells represent a suitable tool for the study of human T cell development and the role of NOTCH expression during maturation of human T cells.

### Lymphoid B, Myeloid and Erythroid Cell Development in Co-cultures with MS5 and MS5/DL1^ind^ Cells

As a first step toward multi-lineage development, sorted CD34^+^CD38^−/low^ CB cells were co-cultured with MS5 and MS5/DL1^ind^ cells in absence of doxycyclin, with or without EPO. As expected from previous works [8], [10], [11], [19], MS5 cells were efficient in supporting multi-lineage CD19^+^ B, CD14^+^/CD15^+^ G/M and GPA^+^CD36^+^ erythroid development (Figure 3A). Mature erythroid cell development depended on EPO addition during culture. Interestingly, MS5/DL1^ind100^ cells did also support similar pluri-lineage development (Figure 3A–C). Differentiation capacities of CD34^+^CD38^−/low^CD45RA^−^CD90^+/−^ cells were similar using MS5 and MS5/DL1^ind100^ cell lines. Two times more CD34^+^ progenitors were detected in cultures of CD90^+^ vs CD90^−^ cells (Figure 3C), compatible with a higher immaturity of CD90^+^ cells [18]. MS5/DL1^ind500^ cells were however less permissive to B and G/M cell differentiation whereas significantly higher erythroid development was observed (Figure 3B and Figure S7). This result was probably related to the leakiness of DL1 expression in this cell line (Figure 1E) that allowed low levels of T cell development (Figure S8) as well as activation of several NOTCH target genes, in absence of doxycyclin (Figure 3D and Figure S9). Moreover, measurements of transcript levels from lineage specific factors indicated that cells developing in contact with MS5/DL1^ind500^ cells had as low Pax5 and cEBPα expression as cells generated in co-cultures with MS5/DL1 cells whereas cells generated in co-cultures with MS5 and MS5/DL1^ind100^ cells had similar levels of Pax5 and cEBPα (Figure 3D), suggesting that low levels of NOTCH activation can seriously interfere with other genetic programs and skew differentiation. Analysis of pro/preB cell generation from CD34^+^CD38^−/low^ CB cells confirmed that low NOTCH expression limits human B cell engagement at a very primitive level (Figure 3E). These latest results led us to focus on the MS5/DL1^ind100^ cells for further multi-lineage culture experiments.

### Multilineage T, B, NK and Myeloid Development in Co-cultures with MS5/DL1^ind100^ Cells

We have previously shown that human T cell potential of immature CD34^+^CD38^−/low^/CD90^+^ cells can be revealed even after 3 weeks of co-culture with MS5, that is the time required for human B cells to develop from such progenitors [8], [10], [11]. In order to allow expression of B and T cell potentials from the same cell population, CD34^+^CD38^−/low^ CB cells were first co-cultured with MS5 and MS5/DL1^ind100^ cells in absence of doxycyclin during 21 days and NOTCH signal was switched on thereafter for T cell development. Analysis of cells generated at 21 days confirmed the presence of CD19^+^ B and CD14^+^/CD15^+^ myeloid cells in co-cultures with both MS5 and MS5/DL1^ind100^ cells with no major difference between cell lines (Figure 4A and B). CD34^+^ progenitors were also detected at this culture time point. Turning on DL1 expression with doxycyclin addition in the culture medium (Figure 1) induced an up-regulation of Gata3, pTα, TCF7 and HES1 transcript levels, indicative of NOTCH pathway activation and induction of T cell differentiation (Figure 4C). Accordingly, keeping NOTCH on for 3 additional weeks by either culturing cells with MS5/DL1 or adding doxycyclin to MS5/DL1^ind^ co-cultures allowed T and NK cell development with detection of DP CD4^+^CD8^+^ T cells or CD56^+^ NK cells at the end of culture period (Figure 4A and B). These results show that T, B and G/M development can be obtained using the DL1 off/on system in MS5/DL1^ind100^ cells.

In the last set of experiments, clonal cultures were performed to test the robustness of our assay (Figure 5 and Figure S10). A total of 300 CD34^+^CD38^−/low^ and 120 CD34^+^CD38^−/low^CD45RA^−^CD90^+^ single cell cultures were initiated in contact with MS5/DL1^ind100^ cells w/o doxycyclin, of which 90 (30%) and 56 (45%), respectively proliferated enough to allow phenotype analysis and further T cell culture at 21 days. Results show that the two tested populations are heterogeneous in terms of potentials, with a variety of clones being identified (Figure S10). CD1a^+^CD7^−^ cells were detected after culture in presence of doxycyclin, probably reflecting the generation of dendritic cells driven by NOTCH pathway (Figure S10) [20]. A high proportion (33 to 66%) of monopotent G/M (CD14^+^ and/or CD15^+^), B (CD19^+^) or T (CD7^+^) clones were identified, whereas multi-potent T/B/G/M cells represented respectively 11,75% and 7% of the proliferating clones in CD34^+^CD38^−/low^ and CD34^+^CD38^−/low^CD45RA^−^CD90^+^ cells (Figure S10) comparable to previous work [10]. In one experiment, the generation of CD56^+^ NK cells was also observed from single cells together or not with T (CD5^+^CD4^+^/CD8^+^) cells, further outlining the diversity of potentials that can be read in this assay (Figure 5). Altogether these results show the relevance of this new experimental system to explore multi-potentiality of single cells from candidate HSC or progenitor cell populations.

## Discussion

In this work, we describe a novel system developed to explore multipotentiality, an essential property of human HSC. This system is based on the mouse stromal cell line MS5, previously used to explore granulo-monocytic and B cell differentiation potentials of human primitive cells [8], [10]. Recently this stromal cell line had also been genetically manipulated to constitutively express the human NOTCH ligand DL1, allowing leukemic blasts from T-ALL patients to proliferate [14] and also normal CD34^+^CD7^−^ human progenitors to differentiate into T cells [13] as originally described for OP9 and S17 cell lines for mouse and human progenitor cells [15], [17], [21]. In accordance with these data, we show that constitutive DL1 expression allow T cell development from immature CB cells up to the DP CD4^+^CD8^+^ stage, a proportion of which expressed also surface CD3. Compared to OP9/DL1 cells [15], [17], [21], human T cell development was weaker after co-cultures with MS5/DL1 cells, due either to a difference in the level of DL1 expression in both cell lines (DL1 expression is higher in OP9 vs MS5 cells probably related to different retro-viral vector constructs used; data not shown) or to the fact that OP9 cells are devoid of M-CSF [22], thus limiting myeloid development at the expense of T cells.

As discussed before, obtaining all differentiation (B/T/Myeloid) potentials to be expressed in a unique well is difficult, due to the incompatibility between molecular pathways activated during engagement to defined lineages. In particular, B and T cell development are mutually exclusive and activation of NOTCH pathway through DL1 or DL4 that is crucial for T cell engagement and differentiation abolishes B cell development [6], [7]. Our working hypothesis was based on a previous study in which we had shown that it was possible first to drive proliferation of single cells and allow them to express their B and myeloid potentials while keeping a T cell potential that could be further tested in a second culture system using xenogeneic FTOC [10]. This experimental system being valuable but circuitous, we designed MS5 stromal cells to conditionally express human DL1 (named MS5/DL1^ind^) upon addition of doxycyclin to the culture medium. We derived three different cell lines MS5/DL1^ind100^, ^ind500^ and ^ind1000^ in relation to the quantity (in ng of p24) of TET/ON lentiviral vector used for the transduction. MS5/DL1^ind1000^ cells were excluded due to detectable DL1 expression in absence of doxycyclin, probably related to leakiness of the conditional expression system. We then showed that T cell development could be reproducibly observed with MS5/DL1^ind100^ and MS5/DL1^ind500^ cell lines in presence of doxycyclin during culture was comparable to the ones obtained in presence of MS5/DL1, in terms of proliferation and differentiation. This result was intriguing, as these cell lines did not express similar levels of DL1, arguing either against a relation between NOTCH expression levels and strong T cell development [23] or for the fact that DL1 surface expression in MS5/DL1^ind100^ cells is already optimum and that an overall higher expression is not necessary to further increase T cell production. In fact, flow cytometry measurement of surface DL1 expression showed that MS5/DL1^ind100^ cells express a wide variety of DL1 levels, very different from the other MS5/DL1^ind^ cell lines. Future experiments in which MS5/DL1^ind100^ cells will be sorted according to low or high levels of DL1 expression will be extremely interesting to fully explore the relation between NOTCH ligand expression levels and hematopoietic cell differentiation. Inversely, washing out doxycyclin during T cell culture led to fast DL1 down-regulation interrupting NOTCH signalling with important effects such as accelerated DP generation and decreased proliferation of such cells were observed in accordance with other studies [12]. Studying the effect of lower NOTCH ligand expression levels on T cell development upon addition/removal of graded amounts of doxycyclin is also an interesting question to address in such TET/on/off system.

In a second set of experiments we tested the ability of MS5/DL1^ind^ cells to support B and myeloid differentiation comparatively to MS5 cells. We observed that MS5/DL1^ind100^ cells were the most interesting cells as they allowed similar differentiation than MS5 cells. MS5/DL1^ind500^ cells were restrictive for B and to a lower extent to myeloid development [24]. Careful analysis of NOTCH target gene expression (*Hes1, Pt*α, *Tcf7, Gata3*) as well as B and myeloid specific transcripts (*Pax5, cEbp*α, *Pu1*) in cultured hematopoietic cells indirectly confirmed that in absence of doxycyclin MS5/DL1^ind500^ cells expressed levels of DL1 that could significantly interfere with early B cell engagement. Decreased production of specific pro- and pre-B cell populations [11] further confirmed this result during co-culture with MS5/DL1^ind500^ cells. Recent data obtained with OP9 cells in which varying levels of DL1 and DL4 expression had been introduced showed that limited levels of DL4, but not DL1, were capable of interference with the B cell differentiation [23]. Although our results are slightly different, it is difficult to compare the respective studies as firstly tested hematopoietic cell populations were different (mouse vs human, cKit^+^Lin^−^Sca1^+^ vs CD34^+^CD38^−/low^ cells), secondly levels of DL1 expression on MS5 and OP9 cells would need to be compared side by side to conclude on the similarity/difference of expression and lastly it is possible that even at similar DL1/4 expression levels MS5 and OP9 cells would not allow similar T cell development, due to other protein/pathway interactions. In any case, it would be very valuable to explore the difference between both cell lines, as well as the effect of conditional DL4 expression in OP9 and MS5 cells on T cell as well as on other lympho/myeloid development for the design and the understanding of better differentiation conditions in human haematopoiesis.

In the last part of our study, we meant to combine both T and B/G/M differentiation protocols in order to obtain multi-lineage development in a single culture dish. Co-cultures of immature cell populations with MS5/DL1^ind100^ cells initiated in absence of doxycyclin and run during 3 weeks allowed B and myeloid cell development as observed with MS5 cells. Interestingly CD34^+^ progenitor cells remained detectable during this culture period. T cell differentiation was promoted following addition of doxycyclin with similar levels obtained to co-cultures using MS5 cells switched to MS5/DL1 cells. These results suggested that testing multi-potentiality of human candidate HSC populations was feasible using this experimental system. In the next step, CD34^+^CD38^−/low^(CD45RA^−^CD90^+^) HSC-enriched populations were tested for their ability to generate T/B/G/M cells *in vitro* at the single cell level. The protocol we developed to assay multipotentiality at the clonal level consisted in dividing proliferating clones at 3 to 4 weeks to look for B and myeloid potentials before to explore the T cell potential. Thus this system resembles the switch *in vitro* cultures we previously designed using MS5 co-cultures and FTOC [10] or could be replaced by switched cultures between MS5 and MS5/DL1 cell co-cultures. However the conditional MS5/DL1^ind100^ cell line is easier and safer as the cell line used is unique and does not require culturing two cell lines in parallel avoiding cross-contaminations. Indeed such accident can be detrimental for B and myeloid cell production prior to T cell development, in particular at the clonal level (JC and FP, not shown).

Results show that single CD34^+^CD38^−/low^(CD45RA^−^CD90^+^) cells generate a wide variety of progeny including multi-potent lympho-myeloid cells. Comparison of results obtained between the tested populations is difficult for they were sorted from different CB samples. Two interesting observations were nonetheless made: first, we could detect dendritic (CD1a^+^/CD7^−^) cells generated from several clones suggesting that other potentials can be studied in this new system, such as erythroid and megakaryocytic cells [25] (upon addition of EPO or thrombopoietin during cultures) and second the frequency of T/B/G/M obtained in this system for CD34^+^CD38^−/low^CD45RA^−^CD90^+^ cells (5%) is similar to the ones we previously obtained testing similar cell population (CD34^+^CD90^+^) in a different experimental setting [10]. Moreover, this frequency is similar to the frequency of SRC measured in this same population using adult [26] and newborn [18] NSG mice supporting the idea that similar cells are read in the *in vivo* and *in vitro* systems. If such speculation is true, then it becomes feasible to screen at the single cell level for specific cell populations using more stringent cell surface markers (such as CD49f, [26]) from different hematopoietic sites [27], in order to refine human HSC phenotype and search for alternate HSC populations, eventually getting close to the mouse situation where HSC are well characterized due to easier and more accurate experimental models.

*In summary*, we have developed a novel experimental system that allows assessment of multi-potentiality from human hematopoietic immature cells. Such assay may represent a valuable tool to refine human HSC populations and could be a complementary and useful approach before animal experimentation. This assay may also open interesting avenues for the follow up on human HSC in strategies developed to improve successful transplantation in humans, such as increasing HSC content during ex-vivo amplification protocols as well as quantifying HSC generated during manipulation of human embryonic stem cells.

## Supporting Information

Figure S1 Comparison of T cell differentiation during cultures with murine MS5/DL1 and OP9/DL1 stromal cell lines. 5000 CD34^+^CD38^−/low^ cells were cultured in contact with MS5/DL1 and OP9/DL1 cells. At the indicated time points, cells were harvested, labeled with anti-human specific antibodies and analyzed by FACS. Shown are results from one experiment.
(TIFF)

Figure S2 Proliferation of human immature cells during T cell differentiation. 5000 CD34^+^CD38^−/low^ were cultured with MS5 cell lines in presence of doxycyclin (1 µg/ml). Cells were counted using trypan blue and FACS analysis at every time point.
(TIFF)

Figure S3 T cell differentiation from CD34^+^CD38^−/low^CD45RA^−^CD90^+^ cells cultured with MS5 cell lines. Cells were labeled with anti-human specifics antibodies and analyzed by FACS after 21 and 42 days of culture.
(TIFF)

Figure S4 Development of human NK cells during culture with MS5 or MS5/DL1^ind^ cells: 10^4^ CD34^+^CD38^−/low^ cells were cultured in triplicates during 42 days in contact with MS5 cells. Human cells were harvested, labeled with anti-CD56 and anti-CD8 antibodies and analyzed by FACS. Shown are results from a typical experiment (A) and from 4 individual CB samples (open circles, B). Median values are indicated as bars.
(TIFF)

Figure S5 T cell development during co-culture of 10^4^ CD34^+^CD38^−/low^ cells with MS5/DL1^ind500^ in presence or not of doxycyclin (1 µg/mL). Cells were stained with anti-human specifics antibodies and analyzed by FACS 21, 35 and 42 days of culture.
(TIFF)

Figure S6 Proliferation capacity of CD7^+^CD4^+^CD8^−^ cells and CD7^+^CD4^+^CD8^+^ cells generated during cultures. 10000 CD34^+^CD38^−/low^ cells were cultured with MS5/DL1 or MS5/DL1^ind^ during 42 days in triplicates. Progeny of such cells were stained with anti-human CD7/8/4 and Ki67 specific antibodies and analyzed by FACS at Day 42 (left histograms). In a second experiment (right histograms), at 35 days of culture, cells were split into cultures with (+doxycyclin or MS5-DL1) or without DL1 (-doxycyclin ou with MS5). Proportion of Ki67+ cells were measured a week later in every gated populations. M&W statistical analysis was used. *, p<0.05; **, p<0.01.
(TIFF)

Figure S7 Multilineage B, G/M and Erythroid cell differentiation of CD34^+^CD38^−/low^CD45RA^−^CD90^+^ cells in co-culture with MS5 or MS5/DL1^ind^ cells without doxycyclin : 10^4^ CD34^+^CD38^−/low^CD45RA^−^CD90^+^ cells were cultured in contact with MS5 stromal cell lines. Cells were harvested after 21 days, labeled with anti-human specific antibodies and analyzed by FACS. Results are from 3 individual CB samples cultured in triplicates. K&W statistical analysis was used. **, p<0.01. ***, p<0.001
(TIFF)

Figure S8 Spontaneous T cell differentiation of CD34^+^CD38^−/low^ cells in co-culture with MS5 or MS5/DL1^ind^ cells without doxycyclin : 15000 CD34^+^CD38^−/low^ cells were cultured in contact with MS5 cells. A. Cells were harvested at 21 days, labelled with anti-human specific antibodies and analyzed by FACS. Results from MS5 and MS5/DL1^ind100^ are from 3–5 CB samples cultured in du- or triplicates. Similar results were obtained with sorted CD34^+^CD38^−/low^CD45RA^−^CD90^+^ cells. B. CD4/CD8 expression levels in cells generated at 42 days of culture. Data from 1 experiment.
(TIFF)

Figure S9 Baseline expression levels of NOTCH target genes in hematopoietic cells co-cultured with MS5 cells in absence of DL1. Progeny of CD34^+^CD38^−/low^ was harvested 7 days after initiating cultures and transcript levels were analysed by quantitative RT-PCR. Results are normalized over ß2m expression levels.
(TIFF)

Figure S10 **A.** Representation by FACS analysis of differents clones obtained from CD34^+^CD38^−/low^CD45RA^−^CD90^+^ cells cultured at clonal level. **B.** Repartition of the proportion of different clones obtained where 120 CD34^+^CD38^−/low^CD45RA^−^CD90^+^ cells were initially cultured, among which 56 (45%) proliferated enough to allow FACS analysis at day 21 and day 42. **C.** Repartition of the proportion of different clones obtained where 300 CD34^+^CD38^−/low^ cells were initially cultured, among which 90 (30%) proliferated enough to allow FACS analysis at day 21 and day 42. Results are the cumulative data from the 2 experiments
(TIFF)
