# Supplementary figures and images for: Assessment of Human Multi-Potent Hematopoietic Stem/Progenitor Cell Potential Using a Single In Vitro Screening System
Source: PLoS One. 2012 Nov 28;7(11):e50495. doi: 10.1371/journal.pone.0050495 (PMC3509091; doi:10.1371/journal.pone.0050495)

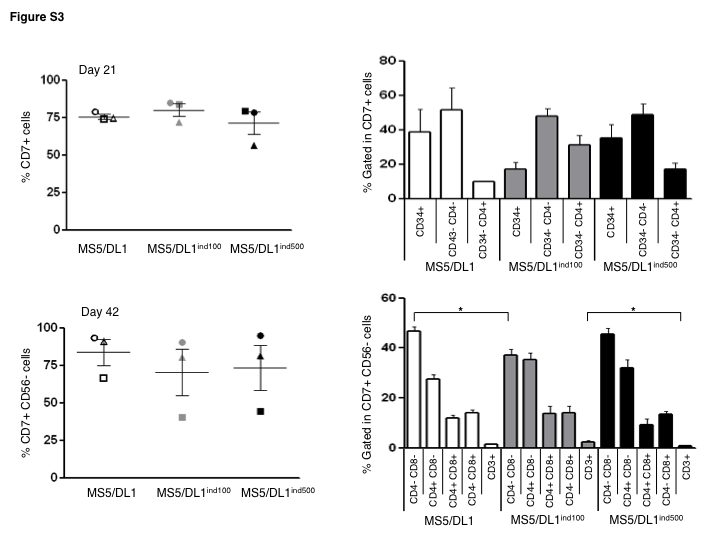

Supplement: Figure S3 — T cell differentiation from CD34+CD38−/lowCD45RA−CD90+ cells cultured with MS5 cell lines. Cells were labeled with anti-human specifics antibodies and analyzed by FACS after 21 and 42 days of culture. (TIFF) [file pone.0050495.s003.tiff]

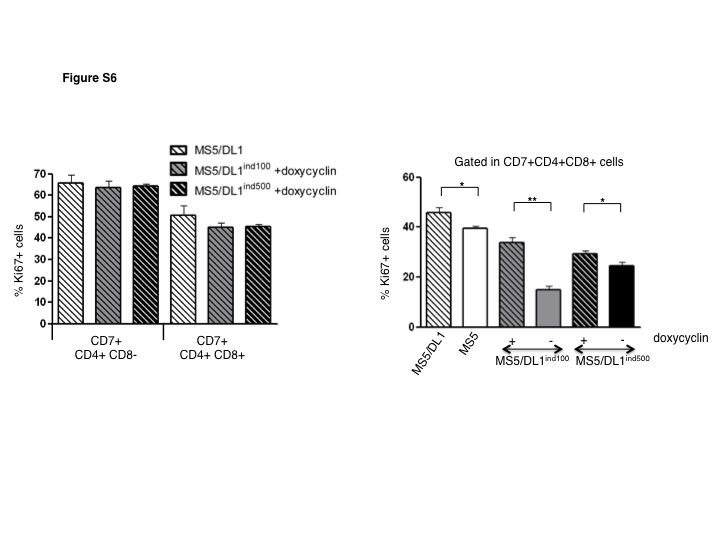

Supplement: Figure S6 — Proliferation capacity of CD7+CD4+CD8− cells and CD7+CD4+CD8+ cells generated during cultures. 10000 CD34+CD38−/low cells were cultured with MS5/DL1 or MS5/DL1ind during 42 days in triplicates. Progeny of such cells were stained with anti-human CD7/8/4 and Ki67 specific antibodies and analyzed by FACS at Day 42 (left histograms). In a second experiment (right histograms), at 35 days of culture, cells were split into cultures with (+doxycyclin or MS5-DL1) or without DL1 (-doxycyclin ou with MS5). Proportion of Ki67+ cells were measured a week later in every gated populations. M&W statistical analysis was used. *, p<0.05; **, p<0.01. (TIFF) [file pone.0050495.s006.tiff]

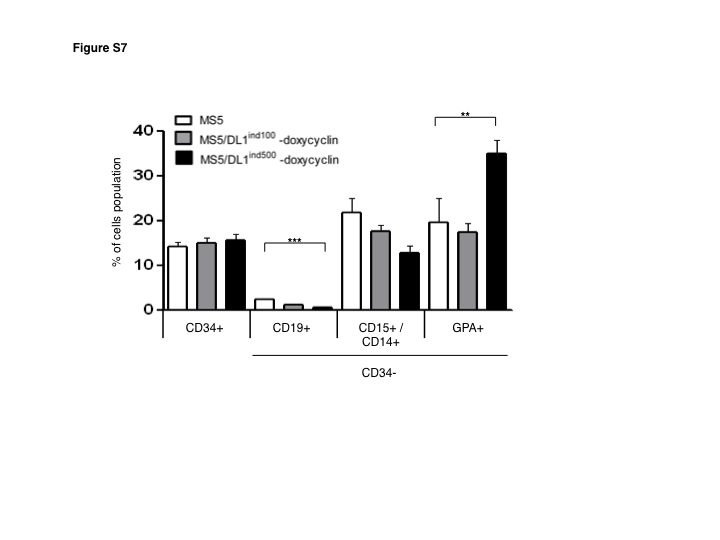

Supplement: Figure S7 — Multilineage B, G/M and Erythroid cell differentiation of CD34+CD38−/lowCD45RA−CD90+ cells in co-culture with MS5 or MS5/DL1ind cells without doxycyclin : 104 CD34+CD38−/lowCD45RA−CD90+ cells were cultured in contact with MS5 stromal cell lines. Cells were harvested after 21 days, labeled with anti-human specific antibodies and analyzed by FACS. Results are from 3 individual CB samples cultured in triplicates. K&W statistical analysis was used. **, p<0.01. ***, p<0.001 (TIFF) [file pone.0050495.s007.tiff]

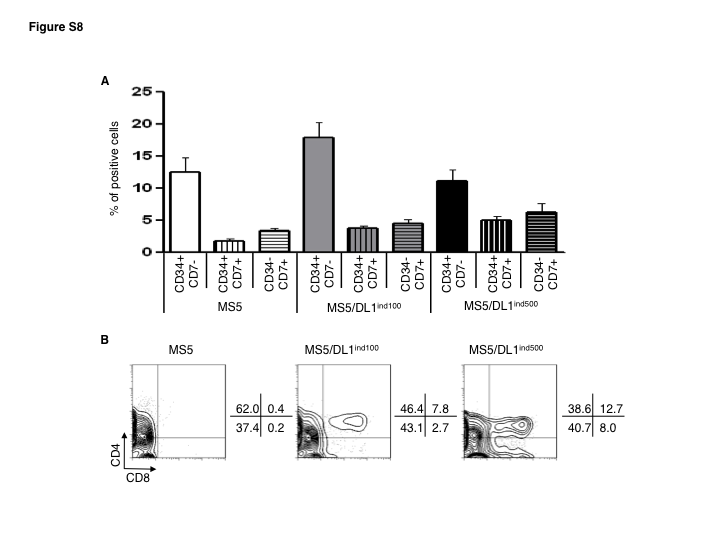

Supplement: Figure S8 — Spontaneous T cell differentiation of CD34+CD38−/low cells in co-culture with MS5 or MS5/DL1ind cells without doxycyclin : 15000 CD34+CD38−/low cells were cultured in contact with MS5 cells. A. Cells were harvested at 21 days, labelled with anti-human specific antibodies and analyzed by FACS. Results from MS5 and MS5/DL1ind100 are from 3–5 CB samples cultured in du- or triplicates. Similar results were obtained with sorted CD34+CD38−/lowCD45RA−CD90+ cells. B. CD4/CD8 expression levels in cells generated at 42 days of culture. Data from 1 experiment. (TIFF) [file pone.0050495.s008.tiff]

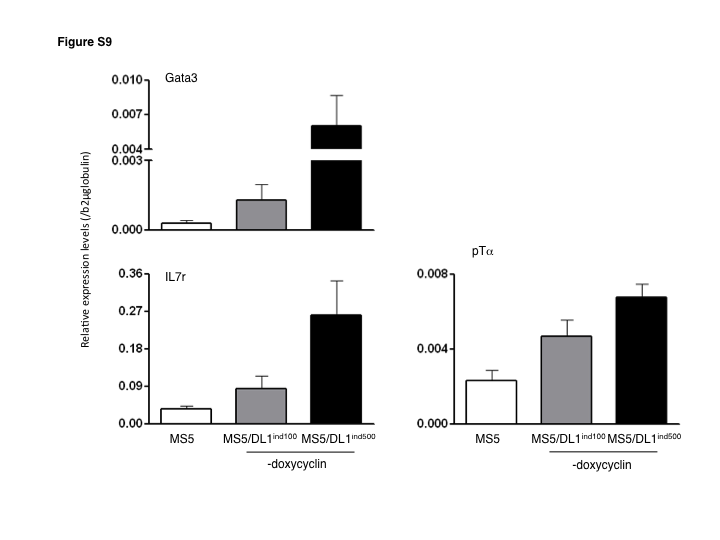

Supplement: Figure S9 — Baseline expression levels of NOTCH target genes in hematopoietic cells co-cultured with MS5 cells in absence of DL1. Progeny of CD34+CD38−/low was harvested 7 days after initiating cultures and transcript levels were analysed by quantitative RT-PCR. Results are normalized over ß2m expression levels. (TIFF) [file pone.0050495.s009.tiff]

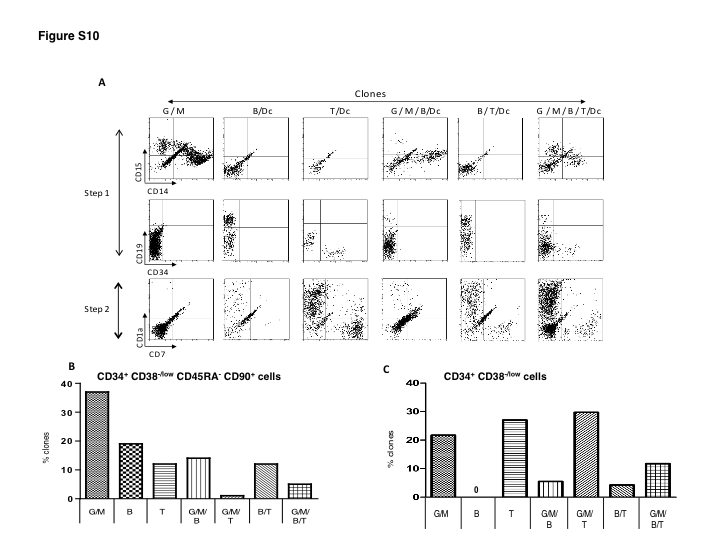

Supplement: Figure S10 — A. Representation by FACS analysis of differents clones obtained from CD34+CD38−/lowCD45RA−CD90+ cells cultured at clonal level. B. Repartition of the proportion of different clones obtained where 120 CD34+CD38−/lowCD45RA−CD90+ cells were initially cultured, among which 56 (45%) proliferated enough to allow FACS analysis at day 21 and day 42. C. Repartition of the proportion of different clones obtained where 300 CD34+CD38−/low cells were initially cultured, among which 90 (30%) proliferated enough to allow FACS analysis at day 21 and day 42. Results are the cumulative data from the 2 experiments (TIFF) [file pone.0050495.s010.tiff]
